# Ankyrin 3: genetic association with bipolar disorder and relevance to disease pathophysiology

**DOI:** 10.1186/2045-5380-2-18

**Published:** 2012-10-01

**Authors:** Melanie P Leussis, Jon M Madison, Tracey L Petryshen

**Affiliations:** 1 Psychiatric and Neurodevelopmental Genetics Unit, Department of Psychiatry and Center for Human Genetic Research, Massachusetts General Hospital, Boston, MA, USA; 2 Department of Psychiatry, Harvard Medical School, Boston, MA, USA; 3 Stanley Center for Psychiatric Research, Broad Institute of Harvard and Massachusetts Institute of Technology, Cambridge, MA, USA

**Keywords:** Ankyrin G, Bipolar disorder, Schizophrenia, Genome-wide association study, GWAS, Axon initial segment, Nodes of Ranvier, GABA, Neurogenesis, Synapse

## Abstract

Bipolar disorder (BD) is a multi-factorial disorder caused by genetic and environmental influences. It has a large genetic component, with heritability estimated between 59-93%. Recent genome-wide association studies (GWAS) using large BD patient populations have identified a number of genes with strong statistical evidence for association with susceptibility for BD. Among the most significant and replicated genes is ankyrin 3 (*ANK3*), a large gene that encodes multiple isoforms of the ankyrin G protein. This article reviews the current evidence for genetic association of *ANK3* with BD, followed by a comprehensive overview of the known biology of the ankyrin G protein, focusing on its neural functions and their potential relevance to BD. Ankyrin G is a scaffold protein that is known to have many essential functions in the brain, although the mechanism by which it contributes to BD is unknown. These functions include organizational roles for subcellular domains in neurons including the axon initial segment and nodes of Ranvier, through which ankyrin G orchestrates the localization of key ion channels and GABAergic presynaptic terminals, as well as creating a diffusion barrier that limits transport into the axon and helps define axo-dendritic polarity. Ankyrin G is postulated to have similar structural and organizational roles at synaptic terminals. Finally, ankyrin G is implicated in both neurogenesis and neuroprotection. *ANK3* and other BD risk genes participate in some of the same biological pathways and neural processes that highlight several mechanisms by which they may contribute to BD pathophysiology. Biological investigation in cellular and animal model systems will be critical for elucidating the mechanism through which *ANK3* confers risk of BD. This knowledge is expected to lead to a better understanding of the brain abnormalities contributing to BD symptoms, and to potentially identify new targets for treatment and intervention approaches.

## Review

Bipolar disorder (BD) is a debilitating illness for which the pathogenesis is poorly understood. BD is defined by alternating episodes of mania and depression. Manic symptoms include impulsivity, high-risk behavior, increased pleasure seeking (hedonia), and decreased sleep, whereas depressive symptoms include anhedonia, impaired cognition, and suicidality [1].

While the biology of bipolar disorder is not well understood, there is a convergence of evidence reviewed elsewhere [2-4] implicating heightened pro-inflammatory processes, specifically increased cytokine production, as well as dysfunction of the hypothalamic–pituitary–adrenal axis, as indexed by enhanced cortisol secretion after dexamethasone or corticotropin releasing hormone challenge. The most consistently reported brain abnormalities in BD include enlarged lateral ventricles and white matter abnormalities, particularly in prefrontal regions. Albeit less consistent, structural imaging studies have found reduced hippocampal volume in BD that is more pronounced in adolescents than adults, possibly due to long-term medication effects, and larger amygdala volume in adults [5]. N-acetylaspartate, a marker of neuronal function, has reduced levels in the dorsolateral prefrontal cortex, anterior cingulate, and hippocampus of individuals diagnosed with BD. Functional neuroimaging studies suggest that activity of limbic regions (hippocampus, amygdala) is increased during emotional processing tasks, while frontocortical activity is decreased during cognitive and emotional tasks.

A number of cellular mechanisms have been implicated in BD pathophysiology and are reviewed in greater detail elsewhere [6]. Of relevance to this article, calcium signaling, which controls many brain essential functions (e.g., neurotransmitter release), appears to be dysregulated in BD based on elevated intracellular calcium concentration in platelets, lymphocytes, and transformed lymphoblasts from patients. A number of intracellular signaling cascades (e.g., brain-derived neurotrophic factor [BDNF] signaling) appear to be perturbed in BD and have been linked to altered glutamatergic neurotransmission, as suggested by altered glutamate levels in plasma, serum, and cerebrospinal fluid from patients, which in turn may impair synaptic plasticity. Mood stabilizers reverse many of the changes described above, providing support for the relevance of these changes to disease. Likewise, the mechanisms of action of BD medications suggest cell biological processes that may be altered in BD [reviewed by [7]]. Lithium has been used for BD treatment for over 60 years, and as such has been extensively studied both clinically and preclinically. Lithium inhibits several enzymes including inositol monophosphatase (IMPase) within the phosphoinositol pathway that mediates many activities, notably cell proliferation and survival [8], as well as glycogen synthase kinase (GSK3) [9] that has a multitude of substrates involved in various cellular processes including cell growth and survival, axonal growth and guidance, synaptogenesis, and neurogenesis [10]. Lithium, as well as the mood stabilizers valproate and carbamazepine, are documented to have neurotrophic and neuroprotective properties, as suggested by larger brain regional volumes in treated BD patients, and upregulation of BDNF and the neuroprotective molecule B-cell lymphoma/leukemia-2 (Bcl-2) in rodent brain. Of note, there is solid evidence that, like antidepressant medications, some mood stabilizers increase adult neurogenesis in rodents in the hippocampus, one of two regions in the mature brain where new neurons are generated [11], suggesting a putative role of adult-born neurons in neural processes underlying BD.

BD has a large genetic component, with increased risk in families of affected individuals, and heritability estimated between 59-93% based on several twin studies [11-15]. In addition, many of the physiological and neural abnormalities discussed above that occur in individuals with BD are also found at higher frequency in unaffected relatives [16], further supporting a genetic basis to this disorder. Given the substantial contribution of genetic factors to BD, identifying the susceptibility genes will unquestionably improve knowledge of the neurobiological underpinnings, which in turn may point to new targets for the development of more effective treatments. However, gene discovery has been extremely difficult, with genetic linkage and association studies fraught with weak and inconsistent results [1,17]. The reasons are many, but primarily small subject samples with low statistical power and a lack of methods to screen genes in a manner unbiased by prior potentially incorrect hypotheses [18]. As reviewed below, recent genome-wide association studies (GWAS) of large subject samples and meta-analyses across multiple studies have been revolutionary in identifying several genes with highly significant and replicated statistical evidence for association with BD. Future GWAS of new subject samples and meta-analyses of the results with existing data will provide increased statistical power to identify additional genes, likely emerging from those falling just below genome-wide significance in current analyses [19]. With compelling candidate risk genes now at hand and others anticipated in the near future, we are entering an era of functional studies to delineate their roles in the normal and diseased brain [20]. Expectations are high that GWAS will lead to major advances in understanding the neurobiological basis of BD. A 2010 Nature editorial entitled “A Decade for Psychiatric Genetics”, highlighted GWAS as one of the new technologies “that are ushering in an era in which the neural circuitry underlying cognitive dysfunctions, for example, will be delineated” [21].

### Genome-wide association studies identify Ankyrin 3 as a bipolar disorder risk gene

GWAS serve as an unbiased approach to identify disease risk genes and pathways in order to understand the underlying molecular and cellular pathophysiology. GWAS test millions of single nucleotide polymorphisms (SNPs) across the genome for differences in frequencies of SNP alleles between case and control subjects. The results require stringent correction for the enormous number of tests, with the genome-wide significance threshold typically set at p < 5 × 10^-8^[22]. Sample sizes in the thousands are required to obtain sufficient statistical power to surpass this significance threshold given the modest effect of any one gene on disease risk. This has been achievable because of collaboration between many research groups contributing DNA samples and/or genotype data into a combined genetic analysis, or for replication of primary findings to obtain imperative support from independent samples that increases confidence in the results.

**Figure 1:**
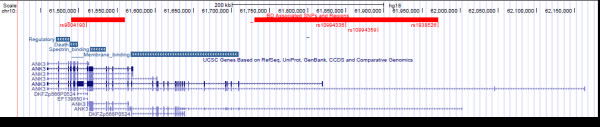
**Human ANK3 gene and protein structure.** The *ANK3* gene has many transcript isoforms (bottom) as a result of extensive alternative splicing of unique 5’ exons containing transcription start sites with up to 43 other exons (exons indicated by vertical bars, introns by horizontal lines). Ankyrin G protein domains (blue bars) are shown above the gene structure. SNPs with evidence for disease association surpassing the genome-wide significance threshold in one or more GWAS of BD or a joint analysis of BD and schizophrenia are indicated at top (red vertical lines). Red bars indicate regions in linkage disequilibrium with the identified SNPs within which the functional sequence variants contributing to disease risk are likely located (5’ associated region on right, 3’ associated region on left). Image adapted from the UCSC Genome Browser.

In 2008, the first gene reported to surpass the genome-wide significance threshold of p < 5 × 10^-8^ in a BD GWAS was diacylglycerol kinase eta (*DGKH*) [23], which has been supported by subsequent studies [24]. This association was particularly appealing since *DGKH* is involved in phosphoinositol signaling through which lithium may mediate its clinical effect [25]. Soon after, a 2009 meta-analysis of three GWAS totaling nearly 4,400 cases and over 6,200 controls identified the ankyrin 3 (*ANK3*) gene with association evidence surpassing the genome-wide significance threshold, and the voltage-gated calcium channel subunit 1c (*CACNA1C*) gene just below the threshold (p = 7.0 × 10^-8^) [26]. Subsequent GWAS and targeted association studies have supported the *ANK3* association, which spans a 250 kilobase region at the 5’ end of the gene (Figure 1; most significant SNPs rs10994336 and rs1938526), as well as indicated a second independent association signal in a 70 kilobase region at the 3’ end (rs9804190) [27-32]. Although several studies used some of the same cases, which may inflate the importance of the *ANK3* results, a meta-analysis of three of these studies reported evidence well above genome-wide significance after removing overlapping subjects (p = 1.1 × 10^-10^) [30]. Some GWAS and targeted studies of *ANK3* have failed to detect significant association surviving multiple test correction with BD risk, age at onset, or psychiatric symptoms, or with risk of other disorders including schizophrenia, major depressive disorder, and attention deficit hyperactivity disorder [24,33-38]. However, many of these studies utilized samples that lacked statistical power to detect small genetic effects such as that of *ANK3*. Subsequent targeted studies also support *CACNA1C* association with BD, as well as schizophrenia and major depressive disorder [39-43], suggesting at least partially overlapping genetic etiology across major mental illness, as also proposed by other studies [44]. Two BD GWAS published in 2011 also reported novel genome-wide significant associations with neurocan (*NCAN*), an extracellular matrix protein involved in neural adhesion and neurite growth [45], lectin mannose-binding 2-like (*LMAN2L*) implicated in protein export from the endoplasmic reticulum, the adjacent genes doublecortin-like kinase 3 (*DCLK3*) and tetratricopeptide repeat and ankyrin repeat containing 1 (*TRANK1*), the prostaglandin F receptor gene (*PTGFR*), and a region on chromosome 3p21.2 containing several genes [27,46]. 

The Psychiatric GWAS Consortium Bipolar Disorder Working Group (PGC-BD) recently published the largest meta-analysis of BD GWAS to date [47]. The primary analysis of 7,481 cases and 9,250 controls from 11 previously published GWAS, some of which are mentioned above, identified two SNPs surpassing the genome-wide significance threshold. The top SNP (rs10994397, p = 7.1 × 10^-9^) is within the 5’ region of *ANK3* that was previously reported, and the other SNP (rs9371601, p = 4.3 × 10^-8^) is located in the *SYNE1* gene*. SYNE1* has an alternative splice form called *CPG2* that functions in postsynaptic recycling of glutamate receptors [48], and has been subsequently associated with major depression [49]. When combining the primary dataset and a replication sample of 4,496 cases and 42,422 controls, both of these results fell just below genome-wide significance. However, two other genes emerged, the previously reported *CACNA1C* (rs4765913, p = 1.52 × 10^-8^) and *ODZ4* (rs12576775, p = 4.4 × 10^-8^), which encodes a member of the tenascin cell surface proteins implicated in neuronal pathfinding [50]. The PGC Bipolar Disorder and Schizophrenia Working Groups also performed a joint GWAS of their primary samples, totaling 16,374 cases and 14,044 controls. Genome-wide significant associations with BD and schizophrenia were detected for three previously reported loci, notably the 5’ region of *ANK3* (rs10994359), *CACNA1C* (rs4765913 and rs4765905), and the chr3p21.3 locus (rs736408 and rs2239547), suggesting they are shared risk factors between BD and schizophrenia.

The GWAS reports have a number of implications. First, as statistical evidence for a particular SNP can fluctuate between samples, genes may rise above or drop below the genome-wide significance threshold in different analyses. It is possible that genes falling below the threshold in a particular analysis are legitimate risk genes, which data from additional samples may help resolve, and that many more genes will be identified in future studies. Second, the genome-wide significant SNPs identified to date have very small effects on disease, with odds ratios under 1.2 on average [23,46,47], indicating an only slightly increased risk of disease for carriers of the SNP allele that is associated with BD compared to non-carriers. It is possible, though, that the contribution to variation in brain processes underlying BD is much larger than for disease risk *per se*. Regardless of the effect size, the genes suggest mechanisms that provide new insight of the neurobiology of BD, and may also reveal new therapeutic targets.

To begin to elucidate the role of *ANK3* in BD, the SNPs identified by GWAS have been examined in relation to brain processes and neuroanatomical abnormalities often linked to BD, as well as for association with other psychiatric disorders. It should be noted that the *ANK3* SNPs have no apparent function, but regardless they serve as markers of the true genetic variants contributing to disease that might be located nearby in the gene. In studies comparing individuals carrying the SNP risk alleles with non-carriers, *ANK3* has been associated with predisposition to anhedonia, altered novelty seeking, impaired threat/stress signal processing, poorer cognition (sustained attention, behavioral flexibility, and working memory), and reduced integrity of white matter tracts [51-55]. These data provide evidence that sequence variation in *ANK3* contributes to functional and structural changes in the brain that may be related to risk for BD. In addition, *ANK3* expression is reported to be lower in superior temporal gyrus of schizophrenia subjects [54], suggesting that *ANK3* downregulation may underlie psychopathology. Given the extent of this evidence for *ANK3* impacting brain function, investigating the neural circuits and processes that it regulates is fundamentally important to understanding the abnormalities underlying BD and perhaps other mental illnesses.

### *ANK3* has essential functions in brain: possible relevance to BD

1) The ankyrin gene family: Ankyrins are a family of membrane skeletal proteins. In mammals, there are 3 ankyrin family members: *ANK1* (encoding ankyrin R), *ANK2* (ankyrin B), and *ANK3* (ankyrin G). *ANK1* is predominantly expressed in erythrocytes, striated muscle, and some central nervous system (CNS) neurons [56]. *ANK2* is mainly expressed in brain, striated muscle, kidney, thymus, and peripheral blood cells [57]. *ANK3* is expressed in nearly all tissues, including brain [58-61].

2) General function and tissue expression of *ANK3:* The ankyrin G protein encoded by *ANK3* has a general role in multiple tissues as a scaffold protein and adapter molecule between various integral membrane proteins and the spectrin cytoskeleton, forming protein complexes that participate in organizing complex microdomains with both extracellular and intracellular functions [For review, see [62,63]]. Ankyrin G is widely expressed throughout the body, including but not limited to heart, skeletal muscle, kidney, erythrocytes, epithelial cells, and brain. In the human brain, *ANK3* is most highly expressed in the frontal cortex, cingulate cortex, hippocampus, thalamus, and cerebellum [64,65]. Importantly, several of these regions are within neural circuits implicated in mood and cognition, processes that are altered in BD.

The function of a gene of interest is typically characterized using transgenic mice in which expression of the gene is increased (i.e., overexpressed) or reduced (i.e., knocked out). In the case of a psychiatric disorder such as BD, examining the behavior of transgenic models may provide insight into relevant neural circuits within which the gene functions. Only one transgenic model of the mouse Ank3 gene has been reported to date, in which brain-specific Ank3 isoforms are exclusively disrupted, while more widely-expressed isoforms are unchanged [66]. The initial characterization of Ank3−/− mice that completely lack brain-specific isoforms noted a progressive early-onset ataxia due to impaired action potential firing at axon initial segments (AIS) of Purkinje neurons in the cerebellum, which is important for motor control [66]. We have found that *Ank3*+/− mice with one functional copy exhibit altered mood-related behaviors and elevated stress reactivity, without any detectable motor deficits as in null Ank3−/− mice. Interestingly, we have found that ankyrin G suppression using viral-mediated RNA interference leads to a highly similar phenotype that can be reversed by chronic lithium treatment, lending credence to the relevance of the behavioral changes to BD (Leussis et al., in press).

3)*ANK3* gene and protein structure: The *ANK3* gene is located within a 700 kilobase region on human chromosome 10 (Figure 1). *ANK3* has several 5’ leading exons containing transcription start sites that are alternatively spliced with 43 downstream exons to generate many transcript variants ranging from 4–15 kilobases in size [59,60]. The functional significance of these unique 5’ exons is not understood, although exon 1b is known to drive transcription of transcript variants that are exclusively expressed in brain, whereas transcripts initiated by other 5’ exons are more widely expressed [66]. In relation to the BD association signals, the 5’ associated region spans exon 1b, and is adjacent to an alternative 5’ exon, exon 1e [26]. The 3’ associated region spans many exons encoding the spectrin-binding and death domains of the ankyrin G protein product [29] (described below).

There is a common molecular organization shared at the protein level across the three ankyrin genes. The N-terminal domain consists of 24 Ank repeats, a known protein binding motif that binds numerous membrane or cytoplasmic proteins [60,67]. These Ank repeats consist of a 33 amino acid structural motif [68]. Following the N-terminal Ank repeats is a spectrin-binding domain that allows ankyrin to link to the cytoskeleton [69]. The binding affinity of both the N-terminal Ank repeats and the spectrin-binding domain is modulated by the C-terminal regulatory region. The very large brain ankyrin isoforms (440 kilodalton [kDa] ankyrin B and 480 kDa ankyrin G) include an extended tail inserted between the spectrin-binding domain and the C-terminal regulatory domain, and are predicted to take an extended random coil shape [59]. Alternative splice variants of the tail domain also give rise to additional isoforms [59]. The function of the tail domain is not yet clear, but it is postulated to play a role in intramolecular interactions with the membrane binding domain that regulate functional interactions [70]. The 480 and 270 kDa isoforms of ankyrin G contain a serine rich domain C-terminal to the spectrin binding domain that appears to be required to restrict them to the axon initial segment (AIS) [71]. While these domains are recognized as functional elements of the ankyrin G protein, several studies have shown the existence of several isoforms of the protein that lack one or more of these domains. Alterations of the domain structure are thought to modulate activity of the protein as described below.

Several large isoforms of ankyrin G have been identified and are the predominant isoforms associated with neuronal function and development. The 440 kDa, 270 kDa (lacks exon 37) and 190 kDa (lacks the serine rich and tail domains) isoforms have been shown to be expressed in neurons [71]. These isoforms are most often associated with the AIS and Nodes of Ranvier, and are required for the organization of these membrane domains. As described below, several studies have suggested lower molecular weight isoforms of ankyrin G lacking most of the membrane binding domain localize to other subcellular compartments. For example, two studies demonstrated that the 100 kDa and 120 kDa isoforms present in mouse macrophages or expressed in 3T3 or COS-1 cells localize to late endosomes and lysosomes involved in protein degradation [72,73]. Furthermore, a 116 kDa (AnkG119) isoform present in kidney and muscle associates with the Golgi apparatus that packages proteins for secretion or transport within the cell [58].

4) Neural functions of *ANK3*.

#### Synaptic organization and stabilization

**Figure 2:**
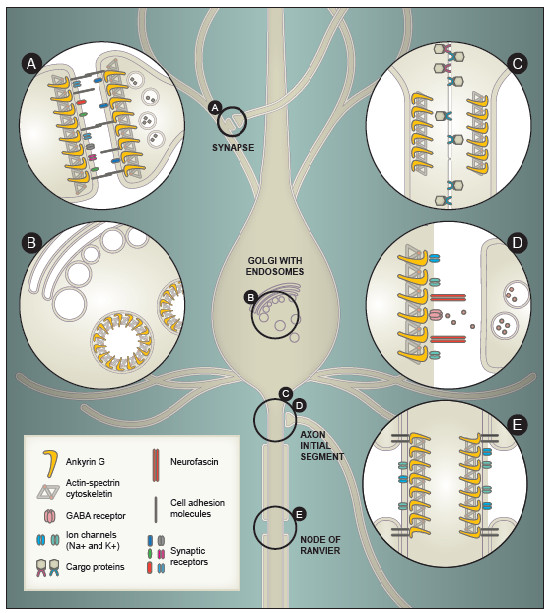
**Known and putative functions of ankyrin G in neurons.** (**A**) Putative scaffolding role at the synapse, where ankyrin G may contribute to the localization of cell adhesion molecules, synaptic receptors, or other synaptic scaffold proteins, as well as to the overall stability of the synapse. (**B**) Some isoforms of ankyrin G localize to late endosomes and lysosomes where they function in cellular trafficking, thereby directing specific proteins to different subcellular regions. In neurons, cellular trafficking occurs at the pre- and post-synapse of neurons, as well as within the cell body as depicted. (**C**) Ankyrin G contributes to cellular compartmentalization, helping to distinguish axonal from dendritic processes through the establishment of an axonal barrier at the axon initial segment (AIS) that prevents transport of non-axonal cargo proteins into the axon. (**D**) Ankyrin G serves as a key scaffold protein at the AIS, interacting with cytoskeletal proteins such as spectrin and actin to localize voltage-gated sodium and potassium channels, cell adhesion molecules (e.g. neurofascin), and GABAergic inhibitory postsynaptic terminals to this region. (**E**) Similar to its role at the AIS, ankyrin G localizes voltage-gated sodium and potassium channels and cell adhesion molecules to the Nodes of Ranvier, which is mediated through reciprocal interactions with myelin-generating glial cells.

Ankyrin G has been implicated in synaptic function (Figure 2A), although the majority of evidence is from studies of the neuromuscular junction (NMJ) in the peripheral nervous system of the fruit fly (*Drosophila*). In *Drosophila*, the presynaptic NMJ is stabilized by giant isoforms of brain-specific Ank2 (Ank2-L), which appear homologous to mammalian ankyrin G large isoforms. These directly bind and organize synaptic microtubules, thus contributing to stability of the presynaptic terminals [74]. Mutations of Ank2-L have been shown to significantly affect NMJ stability in *Drosophila* larva, as evidenced by disintegration of the synaptic cytoskeleton that results in disassembly of presynaptic active zones, withdrawal of synaptic boutons, and reduced terminal size [75]. At the *Drosophila* postsynaptic NMJ, synapse development is dependent on spectrin, which ankyrin directly interacts with, but is also mediated by Ank2-L isoforms [76]. 

There is also evidence that ankyrin G may function in mammalian synapses. For example, ankyrin G has been identified as a component of the postsynaptic density in mouse brain [77,78]. Further, treatment with the mood stabilizer lithium significantly increased ankyrin G levels in the postsynaptic density in rat hippocampus, while valproic acid treatment had a more modest effect on increasing ankyrin G expression [78].

Synaptic defects and reduced synaptic plasticity have been increasingly linked to BD and other psychiatric diseases in both humans and animal models [79,80]. Further, mood stabilizers such as lithium affect the levels of certain synaptic proteins [78,81] and increase long term potentiation (LTP), which is representative of increased neural plasticity [82]. A role of ankyrin G at the synapse, which we postulate occurs in mammals as it has been shown in *Drosophila*, could represent one cellular mechanism of decreased synaptic plasticity that may underlie BD.

#### Cellular trafficking and intracellular signaling

It is postulated that certain isoforms of ankyrin G that lack both the membrane-binding and spectrin-binding domains are associated with Golgi, late endosomes, lysosomes, and the sarcoplasmic reticulum (Figure 2B) that mediate transport and storage of proteins and molecules within cells. For example, in kidney cells, the 116 kDa isoform of ankyrin G localizes with Golgi and endosomes where it is postulated to play a role in organizing microdomains, as well as contributing to transport of polarized vesicles [58,83]. Further, ankyrin G interacts with Hook1, a protein presumed to function in trafficking of proteins to late endosomes [84]. Smaller isoforms of ankyrin G (100, 120 kDa) have also been associated with late endosomes and lysosomes in macrophages [72]. The putative function of these smaller isoforms in trafficking membrane-bound proteins within the cell is as likely to occur in neurons as in other cell types. In fact, endosomal trafficking is essential for neuronal function by targeting proteins to the correct compartments to maintain axo-dendritic polarity, discussed above, and by regulating presynaptic vesicle recycling as well as surface expression and internalization of postsynaptic receptors [85,86].

Ankyrin G is implicated in cellular signaling cascades that mediate a diversity of cellular processes. For example, the small 110 and 120 kDa isoforms in late endosomes and lysosomes have been shown to contribute to lysosome-mediated downregulation of receptors by binding directly to the p85 subunit of phosphatidylinositol 3’-kinase (PI3K). This interaction modulates degradation of the platelet-derived growth factor receptor (PDGFR) that activates different downstream signaling cascades, including the PI3K-Akt and the Ras-MAPK pathways that mediate cellular processes including proliferation and survival [73]. Interestingly, the phosphoinositol pathway is a putative target of lithium and valproate [25,87-89], highlighting a potential overlap between the cellular functions of *ANK3* with BD treatment response.

#### Establishment and maintenance of axo-dendritic polarity

The distinction between dendrites and axons is critical to neuronal function, yet the mechanisms underlying the differentiation of these two compartments are just being identified. Ankyrin G contributes to the maintenance of axo-dendritic polarity of neurons by forming a critical part of the diffusion barrier that assembles in the AIS within 48 hours of axon-dendrite differentiation and acts as a selective filter for axonal transport and diffusion (Figure 2C). When ankyrin G expression is perturbed, the axonal barrier is disrupted and proteins that were not previously detected in the axon are readily observed [90,91]. Additionally, in the absence of ankyrin G, axons lose their identity and gain both structural and molecular characteristics of dendrites, including spine-like protrusions that contain numerous markers for postsynaptic densities, and appear to form synapses, further supporting a role for ankyrin G in regulating axon-defining properties both *in vitro* and *in vivo*[90,92]. Consistent with this function, interactions between ankyrin G and the cell surface protein neuroglian mediate axonal and dendritic morphogenesis, such as the establishment of large dendritic arbors, at least for certain neuronal subtypes in *Drosophila* embryos [93].

Perturbed axo-dendritic polarity could be related to the mechanism of ankyrin G in BD. For neurons to function optimally within neural circuits, they require proper establishment of both axonal and dendritic processes. Interfering in this process, as could occur in individuals with altered levels of functional ankyrin G, would have wide-ranging implications for brain function. This could include alterations in neural circuits involved in mood regulation and cognition that are impaired in BD.

#### Formation and maintenance of the axon initial segment and Nodes of Ranvier

The best-characterized function of ankyrin G in the brain occurs at the AIS and Nodes of Ranvier (NoR) of neurons (Figure 2D, E), where action potentials are generated and propagated down the axon to presynaptic terminals. Ankyrin G is considered a master organizer of the AIS, based on evidence that other AIS-associated proteins, including ΒIV-spectrin, neurofascin-186, and ion channels (especially voltage-gated sodium and potassium channels), depend on the presence of ankyrin G to form localized clusters at the AIS [66,67,94-100]. Further, in hippocampal neuronal cultures, ankyrin G is required for the maturation of the cisternal organelle that functions in regulating calcium levels at the AIS [101]. Recent data from Galiano et al. [102] suggest that ankyrin G is established at the AIS through exclusion of ankyrin G from the distal axon by an ankyrin B cytoskeleton. Subsequent organization of the AIS is orchestrated through multiple ankyrin G protein domains including the membrane-binding, spectrin-binding, and tail domains [71]. Ankyrin G appears to function in this role from early in development through adulthood, suggesting a role in formation and maintenance of the AIS [95]. The disruption of the AIS in knockout mice lacking brain-specific isoforms of ankyrin G correlates with deficits in the initiation of action potentials and decreased repetitive firing in cerebellar Purkinje cell neurons [66]. Recent findings point to a mechanistic role for B-catenin and GSK3-alpha/beta at the AIS, where they contribute to the control of sodium channel density, and hence neuronal excitability [103]. This is interesting given that GSK3 is a known target of lithium [9], suggesting a potential AIS-related mechanism by which lithium may mediate its clinical effect on BD symptoms.

While these studies provide evidence for an essential contribution of ankyrin G to neuronal function, it may also contribute to more dynamic aspects of neuronal homeostatic plasticity. Two studies, one examining rat hippocampal neurons and the other using chick auditory neurons, demonstrated that altered neuronal activity led to changes in the position or length of the AIS, which in turn led to changes in neuronal excitability [104,105]. Such changes could be important to both developmental refinement and function of mature neuronal circuits.

While it is clear that ankyrin G plays a critical role in recruiting and maintaining ion channels at the AIS and NoR, there is also some evidence that ankyrin G plays a modulatory role in the opening or closing of some of these channels. For example, ankyrin G, but not ankyrin B, regulates the inactivation gating of the sodium channel Nav1.6 in cells expressing the human variant of this channel, an effect that is likely mediated by the membrane-binding domain of ankyrin G [106]. Although this effect has only been demonstrated for a single channel type, it is reasonable to postulate that other channels may be similarly modulated by ankyrin G. Altering channel properties can affect neural circuit performance on many levels, thus providing another plausible mechanism through which alterations in ankyrin G levels or function could impact neural circuits involved in BD.

The localization of ankyrin G to NoR is dependent on interaction with glial cells (Figure 2E). Current data suggest that soluble factors secreted by glial cells in both the peripheral and central nervous systems recruit neurofascin-186 (NF-186), which in turn recruits ankyrin G to NoR [107-109]. Glial cells mediate interactions between ankyrin G and the cytoskeleton, thus initiating subsequent recruitment and stabilization of sodium and potassium channels, which are required for saltatory conduction of action potentials along myelinated axons (for review, see [110]).

Alterations in AIS and NoR formation and maintenance, which ultimately affect action potential firing and propagation, have clear implications for proper development and function of neural circuits that may be related to the role of *ANK3* in susceptibility to BD. As evidenced by the ataxia exhibited by knockout mice lacking brain-specific (exon 1b-derived) isoforms of the mouse *Ank3* gene (*Ank3*−/− mice) [66], decreased ankyrin G expression affects neuronal performance to a degree that alters functional output, at least in neural circuits specific to motor control and movement. It is likely that similar deficits, although perhaps less obvious, also occur in other circuits relevant to BD where ankyrin G is expressed. In fact, our research demonstrating altered mood-related behaviors in mice with ankyrin G suppression in the dentate gyrus via RNA interference (Leussis et al., in press) implies that other neural circuits including dentate gyrus are functionally affected by perturbed ankyrin G expression.

Similar to its role in localizing proteins such as ion channels and cell adhesion molecules to the AIS, ankyrin G also directs the localization of inhibitory GABAergic interneuron presynaptic terminals onto the AIS of excitatory neurons (Figure 2D). GABAergic inhibitory activity at the AIS has a critical role in modulating the firing of excitatory neurons in multiple brain regions including the cortex, hippocampus and cerebellum. Conventional knockout of *Ank3* brain-specific isoforms in mice results in disruptions of neurofascin gradients at the AIS of cerebellar Purkinje cells. As a result, GABAergic pinceau synapses from interneurons, which normally localize to the AIS according to the neurofascin gradient, are instead broadly distributed across the axonal and soma membranes, resulting in a disruption of the GABAergic inhibition near the AIS in these mice [111,112]. A similar observation is made for excitatory cortical neurons, which also receive inhibitory inputs from GABAergic interneurons, and are similarly dependent on the presence of ankyrin G for proper localization and distribution of GABAergic terminals at the AIS [113,114]. For a detailed review of the postulated mechanisms underlying this phenomena, see Huang [115].

Although there is no direct evidence for how or if alterations in GABAergic inhibition contribute to BD pathophysiology, several changes in the GABAergic system have been reported in individuals with BD. These include decreased GABA(B) receptors in lateral cerebella [116], and decreased parvalbumin and somatostatin-expressing GABAergic interneurons in the dorsolateral prefrontal cortex [117]. Further, mood stabilizers alter the epigenetic regulation of GABAergic targets, reversing GABAergic gene promoter region hypermethylation that is thought to produce decreased expression of multiple GABAergic targets in BD [118,119]. Thus, the role of ankyrin G in mediating the localization of GABAergic synapses to the AIS could further exacerbate GABAergic dysfunction in BD, as a decrease in GABAergic input would be compounded by improper targeting of inhibitory axon terminals onto excitatory neurons.

#### Neurogenesis and neuroprotective functions

A recent study demonstrated that ankyrin G is required for generation of new neurons (neurogenesis) in the subventricular zone of the adult rodent brain [120]. Ankyrin G is essential for assembly of the subventricular zone niche through lateral adhesion of progenitor cells, which serves as a matrix upon which new neurons are generated. In the absence of ankyrin G, niche assembly does not occur and neurogenesis is substantially reduced or absent. Although this report focused exclusively on neurogenesis in the subventricular/subependymal zone, it is possible that ankyrin G has a similar role in the subgranular zone of the hippocampal dentate gyrus, the other site of neurogenesis in the mature brain.

The modulation of hippocampal neurogenesis in adulthood has been linked to mood disorders such as depression and anxiety, as well as to antidepressant response [For review, see [121,122]]. Further, several mood stabilizers (lithium, valproate, carbamazepine, and lamotrigine) are known to modulate adult neurogenesis in dentate gyrus [11,123], highlighting a putative therapeutic mechanism for these medications. Although few direct links between BD and neurogenesis have been reported, decreased hippocampal volume and altered hippocampal function do occur in BD [5,124] and could result, at least in part, from decreased neurogenesis.

Ankyrin G also plays a protective role in mediating brain immune responses, according to studies in both human and mouse translational models. Specifically, individuals with Alzheimer’s disease that also express high levels of ankyrin G in frontal cortex and elevated levels of ankyrin G antibodies in serum exhibit significantly reduced cognitive decline than individuals with significantly lower ankyrin G serum antibody levels [125]. Further, two different mouse translational models of Alzheimer’s disease that exhibit beta-amyloid accumulation improve following innoculation with ankyrin G antibody, showing reduced brain beta-amyloid pathology [125]. Although this is the first reported occurrence of neuroprotective effects of ankyrin G for a specific brain pathology, it is reasonable to expect that ankyrin G may also act in a neuroprotective fashion in other disease instances in the brain.

### Putative common pathways of *ANK3* and other risk genes in BD pathophysiology

Based on the known functions of *ANK3*, and those of other BD risk genes identified by GWAS discussed above, one can speculate on common pathways underlying these genes that may be related to their mechanism in BD. These pathways are particularly worthy of functional studies in cellular and animal models to delineate the potential role of *ANK3* and other risk genes in BD pathophysiology.

The *CACNA1C* gene encodes the pore-forming alpha 1C subunit of the voltage-gated calcium channel, which is important in mediating neuronal excitability via calcium influx in response to neuronal activity. As ankyrin G is involved in maturation of the cisternal organelle that regulates calcium levels at the AIS [101], both *CACNA1C* and *ANK3* appear to function in calcium-mediated neuronal excitability. Further, an analysis of protein interaction networks found an enrichment of beta adrenergic receptor molecules interacting with *ANK3* and *CACNA1C*[126], implicating both genes in modulation of adenylate cyclase levels via catecholamine binding to beta adrenergic receptors. Adenylate cyclase not only regulates cAMP levels that are important in many intracellular signaling pathways having various cellular effects, but calcium-sensitive adenylate cyclases also enable faster reaction to calcium influx that modulates neuronal excitability. Similarly, the well-documented functions of ankyrin G in localizing inhibitory GABAergic interneuron synapses to the AIS of excitatory neurons, as well as mediating activity-dependent AIS relocation along axons, further supports a common mechanism of *ANK3* and *CACNA1C* in regulation of neuronal excitability.

The *CPG2* splice variant of *SYNE1* functions in turnover of postsynaptic glutamate receptors on excitatory neurons that is important for maintaining and modifying synaptic strength [48]. Ankyrin G has a putative role in synaptic stabilization based on the function of its *Drosophila* homolog [74-76]. Perturbation of ankyrin G or the CPG2 protein could potentially disrupt synaptic transmission within and between neural circuits relevant to BD, leading to the symptoms and cognitive deficits exhibited by patients.

*ANK3* and *DGKH* both appear to participate in intracellular phosphatidylinositol signaling that mediates an enormous diversity of cellular functions, which in the brain include neural cell growth and proliferation, differentiation, and neuroprotection. The ankyrin G isoforms localized to late endosomes and lysosomes bind the p85 subunit of phosphatidylinositol 3’-kinase (PI3K) [73], whose products activate Akt kinase to phosphorylate a variety of protein targets with a range of cellular effects. Diacylglyceraldehyde kinase eta, encoded by *DGKH,* catalyzes the breakdown of diacylglycerol, which is an activator of protein kinase C that, like Akt, has a multitude of targets with diverse effects. Thus, *ANK3* and *DGKH* may both help regulate key kinase proteins in this pathway to modulate a variety of cellular functions. This link between *ANK3* and *DGKH* is particularly interesting as the phosphatidylinositol pathway is a putative target of the both lithium and valproate used in BD treatment [25,87,88,127]. It is therefore possible that sequence variants in *ANK3* and *DGKH* alter the functions of their encoded proteins in this pathway, disrupting downstream neural processes that lead to the emergence of BD symptoms, and that mood stabilizers mediate their clinical effect through normalizing pathway signaling.

A highly speculative link between the *ANK3*, *NCAN*, and *ODZ4* genes is formation of a complex that mediates neuronal migration and axon pathfinding. The neurocan and tenascin-M4 proteins encoded by *NCAN* and *ODZ4*, respectively, are both cell surface proteins expressed in brain that are implicated in these neuronal processes. Given the core function of ankyrin G in coupling integral membrane proteins to the inner membrane cytoskeleton [62,63], ankyrin G may hold tenascin-M4 at the cell surface by binding to the tenascin-M4 intracellular domain. In turn, tenascin-M4 could interact with neurocan on the cell surface, as suggested by the direct binding of neurocan with another member of the tenascin family [128]. Additional evidence for a putative role of ankyrin G in axon pathfinding comes from studies of the ankyrin homolog in the nematode *C. elegans*, unc-44, which is required for proper axon projection to targets [129,130]. Widespread perturbation of axon pathfinding would have global effects on brain function. However, if localized to neural circuits relevant to BD, for example by restricted expression of BD associated genes that mediate pathfinding, the consequence could be a distinct dysregulation of mood and cognition.

## Conclusions

Recent GWAS of BD have provided solid evidence for a handful of genetic risk factors that suggest biological pathways underlying BD and potential new treatment targets, among which *ANK3* is one of the strongest and most replicated genes. The ankyrin G protein encoded by *ANK3* functions as a scaffold protein and adapter molecule between various membrane proteins and the inner membrane cytoskeleton. In the brain, the best characterized functions of ankyrin G include formation and maintenance of the AIS and Nodes of Ranvier, which mediate action potential firing and propagation, and modulation of neuronal excitability. In individuals with BD, altered ankyrin G function in these processes could perturb the proper development and function of neural circuits that regulate mood. Although less studied, ankyrin G is also implicated in adult neurogenesis, synaptic transmission, protein trafficking, and intracellular signaling. Involvement of *ANK3* in biological processes that are shared with other GWAS genes allows speculation about specific BD disease mechanisms, including calcium-mediated neuronal excitability, synaptic transmission, intracellular signaling, neuronal migration, and axonal pathfinding. Functional studies of *ANK3* and other BD risk genes in human populations, as well as animal and cellular models, will be important to elucidate the mechanism by which *ANK3* exerts its effect on BD susceptibility.

## Abbreviations

AIS: Axon initial segment; ANK3: Ankyrin 3; BD: Bipolar disorder; CACNA1C: Calcium channel voltage-dependent, L type, alpha 1C subunit; CNS: Central nervous system; CPG2: Candidate plasticity gene 2; DCLK3: Doublecortin-like kinase 3; DGKH: Diacylglycerol kinase eta; GWAS: Genome-wide association study; kDa: Kilodalton; LMAN2L: Lectin mannose-binding 2-like; NCAN: Neurocan; NMJ: Neuromuscular junction; NoR: Nodes of Ranvier; ODZ4: Odz odd Oz/ten-m homolog 4 (*Drosophila*); PGC: Psychiatric GWAS Consortium; PTGFR: The prostaglandin F receptor gene; SNP: Single nucleotide polymorphism; SYNE1: Spectrin repeat containing nuclear envelope 1; TRANK1: Tetratricopeptide repeat and ankyrin repeat containing 1.

## Competing interests

The authors declare that they have no competing financial or other interests.

## Authors’ contributions

MPL, JMM, and TLP all contributed to the writing of the manuscript. All authors read and approved the final manuscript.
